# Self-Assembled Disulfide Bond Bearing Paclitaxel—Camptothecin Prodrug Nanoparticle for Lung Cancer Therapy

**DOI:** 10.3390/pharmaceutics12121169

**Published:** 2020-12-01

**Authors:** Jingyan Gao, Xiaodong Ma, Lirong Zhang, Jiaqi Yan, Huaguang Cui, Yuezhou Zhang, Dongqing Wang, Hongbo Zhang

**Affiliations:** 1 Department of Radiology Affiliated Hospital of Jiangsu University, Jiangsu University, Zhenjiang 212001, China; 2221813065@stmail.ujs.edu.cn (J.G.); 1000010824@ujs.edu.cn (L.Z.); 2 Frontiers Science Center for Flexible Electronics (FSCFE) & Xi’an Institute of Biomedical Materials and Engineering, Northwestern Polytechnical University, 127 West Youyi Road, Xi’an 710072, China; xiaodong.ma@abo.fi (X.M.); hgcui@mail.nwpu.edu.cn (H.C.); 3 Pharmaceutical Sciences Laboratory, Faculty of Science and Engineering, Åbo Akademi University, FI-00520 Turku, Finland; jyan@abo.fi

**Keywords:** prodrug conjugates, self-assembled, microfluidics, lung cancer, combination therapy

## Abstract

Self-assembled prodrugs (SAPDs), which combine prodrug strategy and the merits of self-assembly, not only represent an appealing type of therapeutics, enabling the spontaneous organization of supramolecular nanocomposites with defined structures in aqueous environments, but also provide a new method to formulate existing drugs for more favorable outcomes. To increase drug loading and combination therapy, we covalently conjugated paclitaxel (PTX) and camptothecin (CPT) through a disulfide linker into a prodrug, designated PTX-S-S-CPT. The successful production of PTX-S-S-CPT prodrug was confirmed by nuclear magnetic resonance (NMR) and high-resolution mass spectrometry (HRMS). This prodrug spontaneously undergoes precipitation in aqueous surroundings. Taking advantage of a flow-focusing microfluidics platform, the prodrug nanoparticles (NPs) have good monodispersity, with good reproducibility and high yield. The as-prepared prodrug NPs were characterized with dynamic light scattering (DLS) and transmission electron microscopy (TEM), demonstrating spherical morphology of around 200 nm in size. In the end, the self-assembled NPs were added to mouse embryonic fibroblast (MEF), mouse lung adenocarcinoma and Lewis lung carcinoma (LLC) cell lines, and human non-small cell lung cancer cell line A549 to evaluate cell viability and toxicity. Due to the redox response with a disulfide bond, the PTX-S-S-CPT prodrug NPs significantly inhibited cancer cell growth, but had no obvious toxicity to healthy cells. This prodrug strategy is promising for co-delivery of PTX and CPT for lung cancer treatment, with reduced side effects on healthy cells.

## 1. Introduction

The mission of medicinal chemists is identifying chemical entities with potential therapeutic utility and value using both computational [1] and synthetic [2] approaches. The successful identification of pharmacodynamically potent compounds does not always ultimately lead to a drug development project. The delivery at the site of action at the appropriate time is a crucial for the active pharmaceutical ingredients (APIs) to fully and properly function, since improper position not only compromises the potency but may lead to unwanted toxicity. Successful drug delivery is often a challenge given the many pharmacokinetic (PK) hurdles it needs to overcome. However, the issue is addressable using a prodrug strategy. More than six decades ago, Albert coined the term “prodrug” [3], referring to compounds bearing little or no pharmacological activity but, after administration, are metabolized to the active parent drug through enzymatic/chemical processes.

APIs, rarely administered alone, are often integrated with one or more non-medical excipients that serve varied and specific pharmaceutical functions. Excipients are crucial to drug delivery in the formulation of stable dosage forms. In general, they have no medicinal properties, and their primary role is to streamline the drug product and facilitate physiological absorption. In some case, excipients dominate the administrable drug formulation while APIs only account for a small. Excipients therefore are often used, which poses some concerns. The outnumbered usage of non-medical excipients is economically inefficient from a manufacturing perspective. The excipients can cause side effects. Taking Food and Drug Administration (FDA)-approved poly(lactic-co-glycolic acid) as an example, its degradation in vivo through hydrolysis of the ester bonds to monomeric lactic acid can lead to the feeling of pain, unexpected allergy, and short-term treatment noncompliance. Therefore, a strategy is needed to lower the proportion of excipients in drug formulations, and even to provide excipients-free drug delivery.

In addition to improving the PK properties of drugs [4], prodrug efforts have expanded drug formulation options [5]. For instance, a piperine prodrug was loaded into hydroxyapatite to obtain a nanoformulation for cancer therapy [6]. The primary advantage offered by prodrugs in terms of drug formulation is the self-assembly into nanomedicines. Compared to excipients assisting with the drug delivery system, the prodrug approach is carrier-free, meaning no extra components are added to the drug formulation except for the prodrug. Chemical modification is a proven approach for designing prodrugs, which conceives of beneficial pharmacokinetic profiles using corresponding parent drugs.

The polymeric drug delivery system, which is the formation of soluble polymer-drug complexes [7] and the recognition between them, depends on the cooperation of noncovalent interactions, so the guest drug is released from the host polymer. The macromolecular prodrugs are polymer–drug conjugates covalently bonded; therefore, the bond breakdown is the first step of drug liberation. Similarly, small drug molecule-based prodrugs can be built both noncovalently [8] and covalently [9]. Recently, the self-assembled prodrugs (SAPDs) strategy has been a focus of interest, due to its carrier-free and self-assembling properties during the formulation processes. The SAPDs can be roughly categorized into macromolecular and small-molecule-based. Macromolecular SAPDs are polymeric units built through the chemical attachment of numerous drug arms to a main chain of synthetic or biological polymer and are frequently spherical in nature. Small-molecule SAPDs consist of a definite number of drug molecules attached to a single promoiety to create a conjugate that are monodisperse in molecular weight.

Prodrug design needs to consider the following: (1) is the drug amendable? (2) what is the need to modify the drug; and (3) will the modification improve the PK profile of the drug?; (4) can the prodrug be readily converted into the parent drug; and (5) can synergistic effects be achieved for specific diseases? The prerequisite of modification is that the drug must possess a synthetic handle that favors reaction with the promoiety that imparts the desired physicochemical improvement. The chemical bond formed through amidation, esterification, and thiol-disulfide exchange reactions were found to be reversible when enzymes or mild chemical conditions were applied. Hence, the prioritized functional groups contain carboxylic acid, amine, hydroxyl, and thiols. Since most anticancer drugs are hydrophobic, their clinical use is limited unless the solubility barrier can be bypassed using some formulations. However, these changes have not provided a satisfactory solution to the problem. Taking commercialized anticancer drug paclitaxel (PTX) and camptothecin (CPT) as examples, patients often suffer from variety of side effects when taxol is administrated and abraxane only has low PTX loading (about 10%). The CPT injection formulation may lead to unexpected hematuria. Therefore, new drug delivery and formulation approaches are needed. To favor self-assembly, drug conjugates need to undergo a change in conditions to induce aggregation/assembly, such as the rapid dilution of a water-miscible organic solvent solution of the prodrug into water. Nanoprecipitation via solvent replacement has been widely used for drug entrapment into nanoparticles (NPs) [10], but combination with other techniques has produced advantages in the nanoprecipitation process. Compared with conventional stirred reactors, nanoprecipitation through microfluidics significantly eliminates batch-to-batch variability [11].

**Figure 1:**
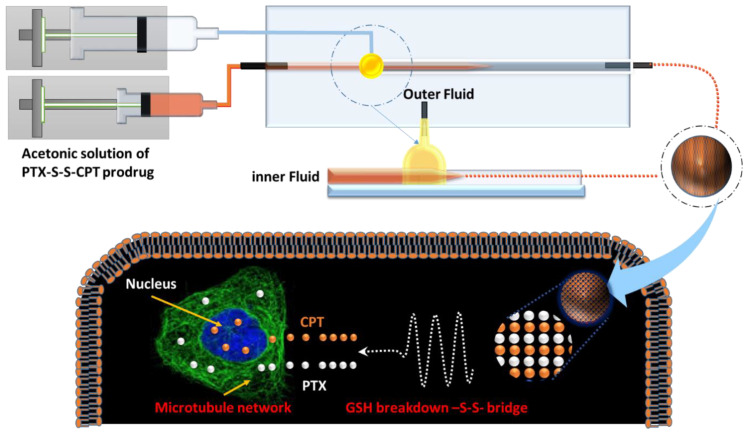
Illustration of camptothecin-S-S-paclitaxel (CPT-S-S-PTX) nanoparticle for lung cancer cell therapy.

Herein, PTX and CPT were selected as prodrug components, since their combination can synergistical cure lung cancer and their current formulation fails to match the demand for efficient co-delivery. Our previous investigation showed that the conjugation of PTX and doxorubicin (DOX) enables self-assembly into NPs [9]. CPT and DOX share a fused multi-benzenic moiety, so a prodrug composed of PTX and CPT will likely also assemble into NPs. However, both PTX and CPT are hydroxyl-groups-bearing compounds, so cannot be directly conjugated together. Based on the evidence that most cancer cells often exist in hypoxia microenvironments, a disulfide-bond-containing linker with dicarboxylic acid handles, which can bond with PTX and CPT, was introduced into the prodrug to respond to the high concentration of glutathione (GSH) in diseased cells [12]. In addition, CPT includes a blueish fluorophore that concurrently provides a means for the visualization of the drug delivery process [13]. Based on above-mentioned considerations, we designed paclitaxel (PTX) and camptothecin (CPT) through a disulfide linker into a prodrug, designated PTX-S-S-CPT, with self-assembling into NPs to fulfill theranostic outcomes for lung cancer. The therapeutic effects of this prodrug NPs were evaluated with the lung cancer cell lines A549, LLC, and healthy mouse embryonic fibroblasts (MEF). As demonstrated in Figure 1, the CPT-S-S-PTX prodrug was fabricated into NPs using our built-in three-dimensional microfluidics platform. The cell uptake and diseased cell growth inhibition were evaluated by co-culturing the lung cancer cell lines Lewis lung carcinoma (LLC), A549, and MEF with as-prepared CPT-S-S-PTX prodrug NPs. We postulated that the NPs can be selectively internalized and accumulated into cancer tissues, then break down into two parent drugs, due to the high-concentration reduced agents.

## 2. Materials and Methods

### 2.1. Materials

Paclitaxel (PTX) and camptothecin (CPT) were purchased from Arisun Chem Pharm Co., Ltd. Xi’an, China). 3,3′-dithiodipropionic acid (DTDP), 4-dimethylaminopyridine (DMAP), and *N*,*N*′-dicyclohexylcarbodiimide (DCC) were purchased from Tansoole (Shanghai, China). DL-dithiothreitol (DTT), acetyl chloride, *N*,*N*-dimethylformamide (DMF), dichloromethane (DCM), and menthol (MeOH) were purchased from Tansoole (Shanghai, China). Cellulose ester membranes (dialysis bag) with a molecular weight cut-off value (MWCO) of 1000 were purchased from Solarbio.com (Beijing, China).

### 2.2. Synthesis of PTX-S-S-CPT

**Figure 2:**
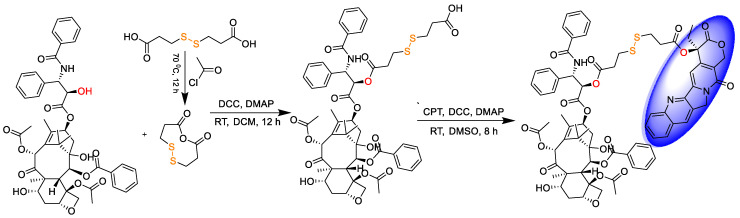
Synthesis route of CPT-S-S-PTX.

As demonstrated in Figure 2, the small molecular drug release system PTX-S-S-CPT was synthesized in three steps under mild conditions. The first two steps were detailed in our previous report [9]; therefore, they are not restated here for producing the intermediate compound PTX-S-S-COOH. PTX-S-S-COOH is then attached to CPT through esterification under the action of DCC and DMAP to obtain final product. In brief, PTX-S-S-COOH (500.00 mg, 0.48 mmol), CPT (199.79 mg, 0.57 mmol), 4-dimethylaminopyridine (11.68 mg, 0.1 mmol), and *N*,*N*′-dicyclohexylcarbodiimide (118.34 mg, 0.57 mmol) were dissolved in DMSO and protected. The reaction mixture was stirred at room temperature overnight, then DMSO was removed by lyophilization. The residues were purified by silica gel column chromatography (MeOH-CH_2_Cl_2_) to obtain yellowish pure products (yield 60%).

### 2.3. Characterization of CPT-S-S-PTX

The structures of the intermediate and final product were characterized by ^1^H nuclear magnetic resonance (NMR) and electrospray ionization mass spectrometry (ESI-MS), and the results showed that we successfully produced prodrug CPT-S-S-PTX. The ^1^H NMR spectra of DTDP, DTDPA, CPT, PTX, PTX-S-S-COOH, and CPT-S-S-PTX were recorded on a Bruker 500 NMR spectrometer (Bruker, Billerica, MA, USA) and chemical shifts (*δ*, ppm) are reported relative to the residual solvent peak. Chemical shifts (*δ*, ppm) are reported relative to the solvent peak (CDCl_3_, 7.26 [1H]; DMSO-*d*_6_, 2.50 [1H]). Mass spectra were recorded on a Bruker Daltonics microTOF-Q mass spectrometer (Bruker, Billerica, MA, USA). The FTIR spectra of PTX, CPT, PTX-S-S-COOH, and PTX-S-S-CPT were recorded on a Thermo Scientific Nicolet iS50 Fourier transform infrared spectrometer (Waltham, MA, USA) in the wavenumber range of 400–4000 cm^−1^.

### 2.4. Assembly of Three-Dimensional Microfluidic Chip

We previously described the production of a three-dimensional microfluidic co-flow focusing device [14]. In brief, two borosilicate cylindrical glass capillary (World Precision Instruments Ltd., Sarasota, FL, USA) assemblies were mounted on a transparent glass slide. One side of the inner capillary (outer diameter of around 1000 μm) was tapered by a capillary puller (P-31, Narishige Co., Ltd., Tokyo, Japan). The tapered capillary was polished using sandpaper (Indasa Rhynowet, Shandong, China), until the cross-section of the tapered end remained flattened. Then, the well-tailored inner capillary was inserted into the truncated outer capillary with an inner diameter of about 1120 μm, and coaxially aligned. A transparent bicomponent epoxy resin (5 Minute^®^ Epoxy, Devcon, Shenzhen, China) was used to glue the capillaries where required.

### 2.5. Preparation of Nanoparticles

Firstly, CPT-S-S-PTX prodrug particles were prepared in flask-based reactors. In brief, the acetone prodrug solution was added dropwise into water with vigorous stirring, followed by centrifugation to remove acetone and resuspension of the particles. The NPs were also fabricated using our in-house microfluidics devices. The acetone solution of CPT-S-S-PTX was filled into a 2 mL syringe that served as the inner flow, while the outer flow was 0.1% Pluronic^®^ F-127 (Sigma-Aldrich, Shanghai, China) aqueous solution (Figure 1) that was filled in a 40 mL syringe. Two flows were separately infused by pumps (LSP01-1A, Longer Precision Pump Co., Ltd., Shanghai, China) into the capillary of the microfluidic device at a constant flow rate through polyethylene tubes, so that the inner fluid was focused by the outer continuous fluid in the microfluidics chip. During the mixing of the two flows, CPT-S-S-PTX self-assembled into NPs due to its migration from the acetone to water. To optimize the physicochemical properties of the prepared NPs, including particle size, polydispersity index (PDI), and ζ-potential, several process variables and formulation parameters, including the flow ratio between the inner and outer fluids and the concentration of CPT-S-S-PTX, were evaluated.

### 2.6. Characterization of the Nanoparticles

The size of nanoparticles and surface ζ-potential were investigated using dynamic light scattering with a Zetasizer Nano ZS (Malvern Instruments Ltd., Marvin, UK). For individual measurement, the sample (1.0 mL) was placed in either a disposable polystyrene cuvette (SARSTEDT AG & Co., Nümbrecht, Germany) or a capillary cell (DTS1070, Malvern, Marvin, UK). Both the size and ζ-potential were measured in triplicate. The structure of the fabricated NPs was viewed using a transmission electron microscope (TEM; JEOL 1400 Plus, JEOL, Tokyo, Japan) at an acceleration voltage of 120 kV. The TEM samples were prepared by dropping 10 μL of the nanoparticle suspensions (about 1.0 mg mL^−1^) onto carbon-coated copper grids (200 mesh; Ted Pella, Inc., Redding, CA, USA). The liquid was blotted away after 5 min of sample mounting, then air-dried prior to imaging.

### 2.7. In Vitro Release of PTX and CPT from the Nanoparticles

The cumulative levels of PTX and CPT released from the CPT-S-S-PTX nanoparticles were characterized using the dialysis method [15]. In brief, 5 mL of CPT-S-S-PTX nanoparticle suspension (1.0 mg mL^−1^) in phosphate-buffered saline (PBS; 1×, pH 7.4, used as before except for additional mention) was transferred to a dialysis membrane bag (MWCO 1000, Fisher Scientific). The dialysis system was suspended in a release volume of 50 mL PBS at 37 °C with or without DTT (10 mM), and immersed in a water bath at 37 °C with horizontal disturbance. Herein, DTT was used as a reducing agent to simulate the existence of GSH in cancer cells. At predetermined time interval, 50 μL of release medium was collected for the HPLC assay [16]. An equal volume of the above-mentioned PBS at the same temperature was added immediately to maintain constant release volume. The s of PTX and CPT were quantified by HPLC using an Agilent 1100 system (Agilent Technologies, Altadena, CA, USA). The released PTX and CPT were identified using a Waters Symmetry Shield RP18 Column (4.6 × 250 mm, 5 mm, Waters Corporation, Milford, MA, USA) as the stationary phase, and the wavelength of detection was 254 nm. The mobile phase consisted of solvent A (Milli-Q water with 0.1% trifluoroacetic acid (TFA)) and solvent (acetonitrile with 0.1% TFA), was infused through the column at a flow rate of 1.0 mL/minute. A gradient of 5–95% of solvent B over 20 min within a 25 min running time was applied to first identify the retention time of the parent drugs (Figure S1), then, we derived a linear formulation between the known concentrations of the two parent drugs and the corresponding integral under the curves (Figure S2). The unknown quantity of drugs in the release medium was determined according to the formulation.

### 2.8. Cell Assays

#### 2.8.1. Cell Culture and Maintenance

Mouse embryonic fibroblast (MEF), mouse lung adenocarcinoma cell line LLC, and human non-small cell lung cancer cell line A549 were used for in vitro studies. The LLC and MEF cells were cultured in high-glucose Dulbecco’s modified eagle medium (DMEM) supplemented with 10% fatal bovine serun (FBS), 1% penicillin–streptomycin, and 2 mM L-glutamine, and the A549 cells were maintained in F12K Kaighn’s modification medium (F12K; Gibco Laboratories, Grand Island, NY, USA) with 10% FBS at 37 °C in a humidified incubator with 5% CO_2_. Cells were passaged 2–3 times a week once they reached 90–100% confluency. These cells were kindly provided by the Stem Cell Bank, Chinese Academy of Sciences.

#### 2.8.2. Cytotoxicity Assay

To estimate the drug efficacy in cancer and healthy cells, we measured the cell viability using a cell counting kit-8 (CCK-8; Dojindo, Kumamoto, Japan) according to the manufacturer’s instructions. Briefly, LLC, A549 cancer cells, and MEF cells were seeded into 96-well plates at a density of 2000 cells/well. After adhering to the wall, cells were treated with 5 nM, 10 nM, 25 nM, 50 nM, 100 nM, and 500 nM free drug or an equal amount of SAPD suspension and incubated for 48 h. Free drugs were all suspended in DMSO and the concentration of DMSO was no more than 0.1%. At particular points in time, 10 μL of CCK-8 solution was added into each well for 4 h. The absorbance was detected by the Benchmark Plus™ Microplate Spectrometer (Bio-Rad Laboratories Inc., Hercules, CA, USA) at 450 nm.

#### 2.8.3. Cellular Uptake Evaluation

Cells were incubated with CPT and NPs for 24 h at 37 °C in 24-well plates, on which we placed a cover class on each well. The final concentrations of CPT and PTX-S-S-CPT were 50 nM. After incubation, cells were washed twice with PBS and then fixed with 4% paraformaldehyde for 30 min at room temperature. Then, we removed the cover glass and placed it upside down on the microslide using tweezers. The cellular fluorescence images were recorded using a Nikon C2+&N-SIM E microscope (Nikon Corporation, Tokyo, Japan).

## 3. Results

### 3.1. Characterization of CPT-S-S-PTX Prodrug

**Figure 3:**
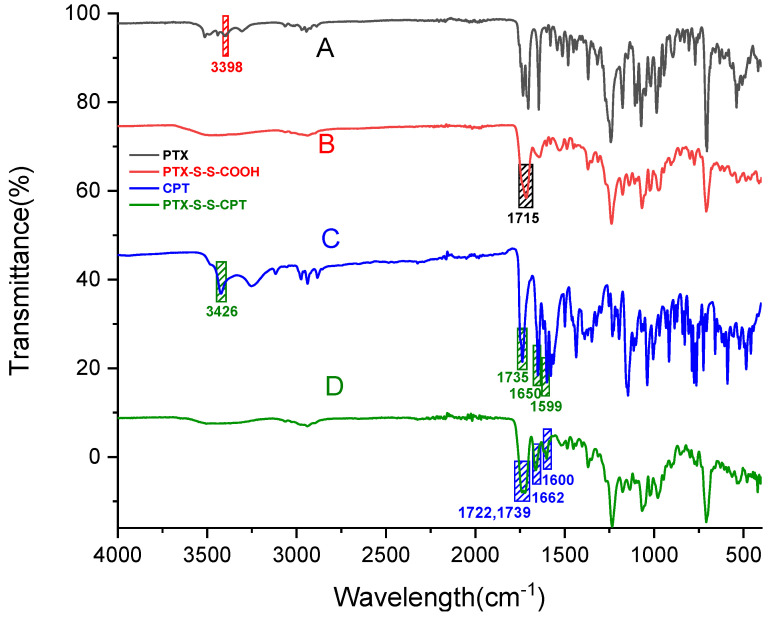
FTIR spectra of (**A**) paclitaxel (PTX); (**B**) PTX-S-S-COOH; (**C**) camptothecin (CPT); (**D**) CPT-S-S-PTX.

The FTIR spectra of PTX, CPT, PTX-S-S-COOH, and CPT-S-S-PTX are presented in Figure 3. As shown in Figure 3A, the characteristic –OH stretching vibration peaks of PTX (Figure 3B) occurred at 3398 cm^−1^. An intense peak was observed at 1715 cm^−1^ (Figure 3B), which indicates the absorption of the C=O of carboxylic acid and suggests the successful synthesis of PTX-S-S-COOH. The position of the peaks at 3426 (–OH), 1735 (ester), 1650 (C=O), and 1599 (C=C) are associated with the feature of CPT in Figure 3C. In comparison, the absence of the –OH band and redshift of bands 1722, 1739, 1662, and 1600 in Figure 3D imply the production of the desired prodrug conjugate. To avoid the unwanted dimeric intermediate PTX-S-S-PTX, DTDP was dehydrated by refluxing in methylene chloride at 70 °C to obtain cyclic anhydride DTDPA first; DTDPA was then reacted with PTX to produce only monomeric PTX-S-S-COOH.

**Figure 4:**
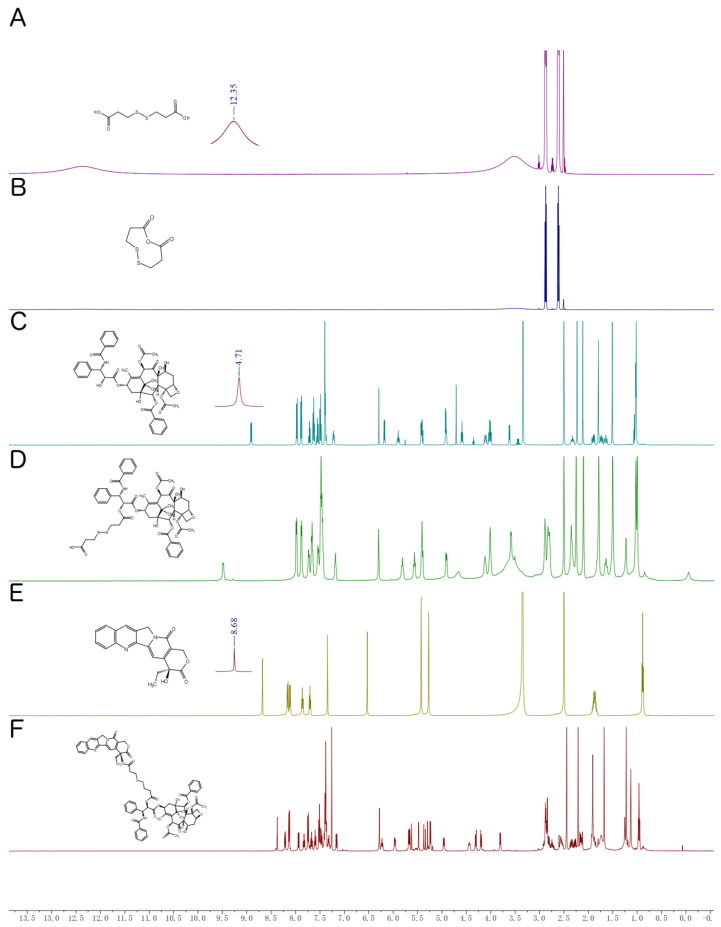
^1^H nuclear magnetic resonance (NMR) spectra of (**A**) 3,3′-dithiodipropionic acid (DTDP) in DMSO-d_6_; (**B**) 3,3′-dithiodipropionic acid anhydride, (DTDPA) in CDCl_3_; (**C**) PTX in CDCl_3_; (**D**) PTX-S-S-COOH in DMSO-d_6_; (**E**) CPT in DMSO-d_6_; (**F**) PTX-S-S-CPT in CDCl_3_.

The ^1^H NMR spectra of CPT-S-S-PTX and its intermediates are shown in Figure 4. Figure 4A–D depicts the chemical shift of DTDP, DTDPA, PTX, and PTX-S-S-COOH proton, respectively, which were reported in our previously paper [9]; therefore, no further statement is provided here. The chemical shifts of CPT-S-S-PTX of the proton are shown in Figure 4F. ^1^H NMR (500 MHz, Chloroform-*d*) δ 8.38 (s, 1H), 8.21 (d, *J* = 8.5 Hz, 1H), 8.13 (d, *J* = 7.2 Hz, 2H), 7.94 (d, *J* = 8.2 Hz, 1H), 7.85–7.79 (m, 1H), 7.74 (d, *J* = 7.3 Hz, 2H), 7.67 (t, *J* = 7.5 Hz, 1H), 7.60 (t, *J* = 7.4 Hz, 1H), 7.51 (t, *J* = 7.7 Hz, 2H), 7.47 (d, *J* = 7.4 Hz, 1H), 7.42–7.36 (m, 7H), 7.16 (d, *J* = 9.2 Hz, 1H), 6.28 (s, 1H), 6.23 (t, *J* = 7.9 Hz, 1H), 5.96 (dd, *J* = 9.2, 3.3 Hz, 1H), 5.74–5.59 (m, 3H), 5.48 (d, *J* = 3.4 Hz, 1H), 5.36 (d, *J* = 17.2 Hz, 1H), 5.29 (s, 1H), 5.24 (d, *J* = 6.7 Hz, 2H), 5.01–4.90 (m, 1H), 4.50–4.39 (m, 1H), 4.31 (d, *J* = 8.4 Hz, 1H), 4.20 (d, *J* = 8.5 Hz, 1H), 3.80 (d, *J* = 7.0 Hz, 1H), 2.93–2.80 (m, 8H), 2.53 (dd, *J* = 9.4, 6.6 Hz, 1H), 2.44 (s, 3H), 2.26–2.37 (m, 3H), 2.21 (s, 3H), 2.17–2.12 (m, 2H), 1.91 (s, 3H), 1.24 (d, *J* = 14.3 Hz, 6H), 1.13 (s, 3H), 0.96 (t, *J* = 7.5 Hz, 3H).

The PTX-S-S-COOH most likely reacted with the CPT functional hydroxyl group, whose proton chemical shift occurred at 8.68 ppm (Figure 4A), but the peak disappeared in Figure 4F after their conjugation. The observed mass of 1398.4103 [M + Na]^+^ (Figure S3) from the HRMS spectra also suggested the successful synthesis of PTX-S-S-COOH with CPT into the prodrug CPT-S-S-PTX.

### 3.2. Characterization of Prodrug-Assembled Nanoparticles

**Figure 5:**
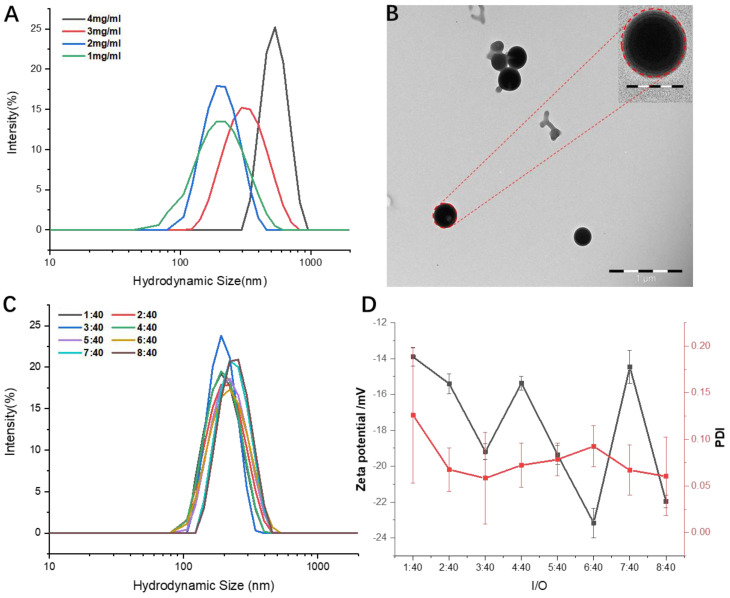
Characterization of PTX-S-S-CPT nanoparticles: (**A**) hydrodynamic diameters (*D*_H_) of prodrug nanoparticles at different concentrations; (**B**) transmission electron microscopy (TEM) images of assembled prodrug nanoparticles (NPs) at the prodrug concentration of 2 mg/mL. Scale bar, 1 μm; inset images with scale bar of 200 nm; (**C**) hydrodynamic diameters (*D*_H_) and (**D**) zeta-potential and polymer dispersity index (PDI) of as-prepared NPs at different inner/outer (I/O) fluid flow with the prodrug concentration of 2 mg/mL.

After the injection of the acetone solution of PTX-S-S-CPT into an aqueous solution, the prodrug formed into uniform spherical particles. Using microfluidics devices, the concentration of prodrug and inner/outer (I/O) fluid flow were tuned to optimize the morphology and size of the prodrug NPs. As shown in Figure 5A, higher prodrug concentrations correspond to bigger NP sizes at the fixed I/O fluid flow of 2/40, but concentration showed no relationship with polydispersity index (PDI). Noticeably, a 4 mg/mL prodrug input achieved more well-distributed NPs than others, whereas 3 mg/mL prodrug produced the worst PDI. When the prodrug concentration was set to 2 mg/mL, the size of the obtained NPs was recorded through the dynamic light scattering (DLS) analysis as about 200 nm (Figure 5A) with a spherical shape (Figure 5B). Under the same prodrug concentration of 2 mg/mL, changing the I/O fluid flow from 1:40 to 8:40 had no significant effect on the prepared NPs size, ranging from 190 to 280 nm (Figure 5C); the PDI changed from about 0.06 to 0.12, but the ζ-potential fluctuated between −14 and −23 mV (Figure 5D).

### 3.3. In Vitro Release of CPT from the CPT-S-S-PTX Nanoparticles

**Figure 6:**
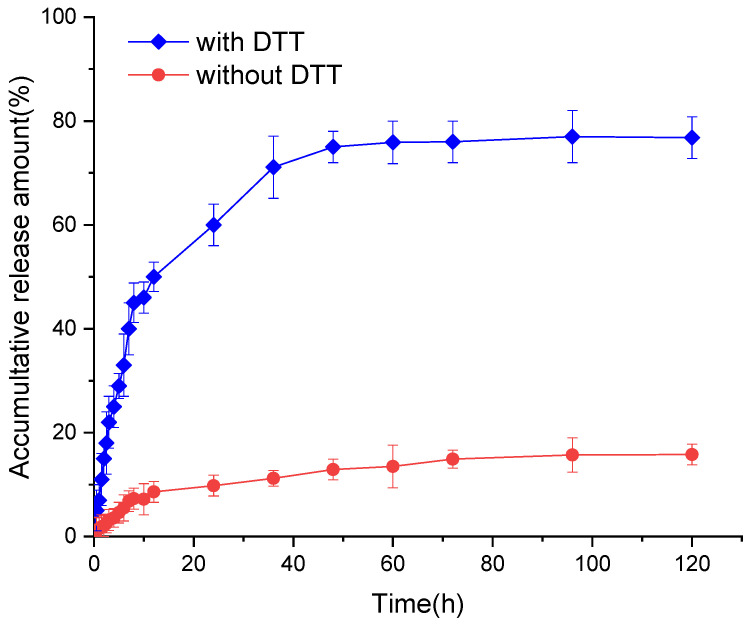
The release of CPT from CPT-S-S-PTX.

Figure 6 depicts the in vitro release behavior of the CPT-S-S-PTX prodrug NPs with and without DTT. The CPT liberation from the CPT-S-S-PTX prodrug NPs showed burst release features then slow increases, finally reaching a plateau. Although the overall release propensity under the two condition was consistent, we observed a significant difference in the presence of DTT, which remarkably stimulated the drug release. In detail, from the first 10 h, about 8% of CPT was liberated when DTT is not added, whereas more than 43% of CPT was released when DTT was introduced. About 13 h were required to release 50% of CPT in reduced conditions, whereas only 10% of CPT is release within 120 h when the solution was DTT-free. The incomplete parent drug release was lower; less than 80% of CPT release was detected. The release of CPT and PTX depends on the break of disulfide linker and hydrolysis of the ester bond that bridges CPT and PTX.

### 3.4. In Vitro Cell Viability Assay

**Figure 7:**
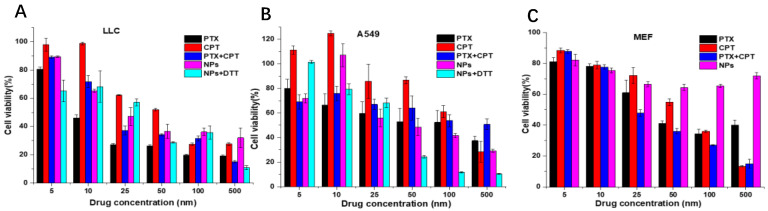
Cytotoxicity of PTX, CPT, and PTX+CPT mixture in equal amounts NPs and NPs+dithiothreitol (DTT) against (**A**) Lewis lung carcinoma (LLC), (**B**) A549, and (**C**) mouse embryonic fibroblast (MEF) cell lines within 48 h.

The cell toxicity of free drugs (CPT, PTX, and CPT+PTX in equal amounts), CPT-S-S-PTX prodrug NPs alone, and NPs with DTT was investigated against LLC, A549, and MEF cells based on the CCK-8 assay. We found that the viabilities of all the above-mentioned cell lines were reduced to different degrees when co-incubated with the experimental reagents. For free CPT, PTX, and the physical mixture of equal molar amounts of CPT plus PTX, a dose-dependent cell proliferation was consistently observed, indicating that the cytotoxicity of free CPT, PTX, and prodrug NPs to cancer cells is concentration-dependent (Figure 7A,B). Conversely, the viability of MEF cell lines remained above 70% viability even when 500 nM NPs were applied (Figure 7C), which implied the prodrug NPs selectively inhibited cancer cell proliferation.

### 3.5. Cell Uptake

**Figure 8:**
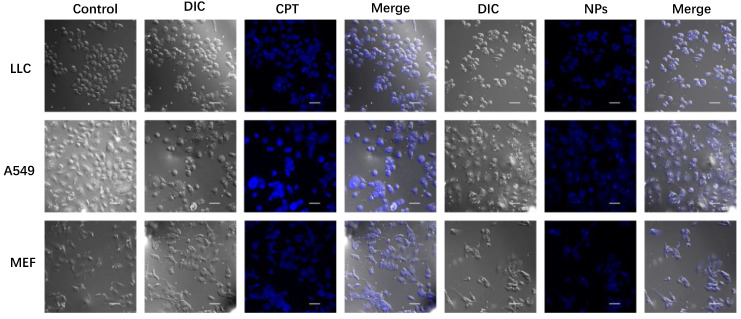
Confocal microscope image of LLC, A549, and MEF cell lines when incubated with CPT and CPT-S-S-PTX prodrug NPs. Scale bar = 50 μm. The control group was the cells without drug treatment. The drug- and nanoparticle-treated cells are presented as images in bright field channel (DIC), 4′,6-diamidino-2-phenylindole (DAPI) channel (405 nm, blue), and merged.

The cellular uptakes of CPT and CPT-S-S-PTX prodrug NPs for LLC, A549, and MEF cells were evaluated using a confocal microscope with the 4′,6-diamidino-2-phenylindole (DAPI) channel and a 405 nm excitation wavelength. As illustrated in Figure 8, NPs exhibited high uptake in cancer cells LLC and A549, whereas lower internalization was observed in healthy cells; the staining of free drug CPT was barely observable in all tested cell lines. Although the incorporation of PTX into prodrug does not affect cellular uptake of CPT, the fluorescence absorption of prodrug NPs was somewhat quenched. We observed that the cellular skeleton of NPs-treated cancer cells became blurred and indistinct compared with the control, implying that cell apoptosis had occurred.

## 4. Discussion

The anticancer function of PTX, a natural product derived from *Taxus brevifolia*, a slow-growing and rare evergreen found in the old-growth forests of the Pacific Northwest, was discovered; it remains the first-line treatment for a variety of tumors, including ovarian, breast, lung, cervical, and pancreatic cancer, given its role in the block assembly and disassembly of cell microtubule polymerization dynamics [17]. The main challenge limiting its broader application spectra is its poor solubility, which makes it hard to transport into cells across the cell membrane since the majority of drugs are deposited in aqueous solution. Currently, two generations of PTX medications, taxol [18] and abraxane [19], have been successfully marketed. However, some side effects, such as low patient compliance, due to possible allergy and low drug loading confined by fabrication approaches, has called for the creation and exploitation of new PTX-based medicines. Pei et al. reported a theranostic abraxane-like prodrug formulation that has a PTX dimer linked with a thioether linker (PTX_2_-S), and has photosensitizer IR780 iodide as the core and human serum albumin (HSA) as the stealth shell, which increased drug loading from 6.6 to 48.7 wt % compared to abraxane, and realized spatiotemporal hyperthermia under light irradiation [20]. CPT is also a botanic anticancer drug that is thought to be a potent topoisomerase inhibitor that interferes with the function of topoisomerase in DNA replication. CPT binds to the topoisomerase I and DNA complex, resulting in a ternary complex, stabilizing it and preventing DNA ligation, thereby causing DNA damage [21]. Structurally, CPT is composed of four fused aromatic rings and forms a planar skeleton, leading to its water insolubility. This heterocyclic feature creates a UV-excitable chromophore in CPT [22], which enables drug tracking and imaging. CPT [23] and taxanes are chemotherapies that have shown significant activity against small-cell lung cancer. For more than two decades, combination chemotherapy has been the standard treatment for patients with small-cell lung cancer. Hence, combining these two compounds to produce synergic results is logical, providing the motivation for this study.

Following our previous synthesis route [9], the CPT-S-S-PTX prodrug was produced. CPT-S-S-COOH can be first synthesized and then conjugated with PTX. We tried both methods, but CPT-S-S-COOH is poorly dissolved in the commonly used eluents, DCM and MeOH. To facilitate the purification process, we decided to produce PTX-S-S-COOH instead. For the nanofabrication of the prodrug microfluidics platform, the robustness of this approach was fully demonstrated by the easily adjustable/controllable parameters, the continuously obtained products, and, most importantly, the well-controlled product quality, highlighting the advantages of microfluidics nanoprecipitation over other solvent replacement approaches. The key module for fabricating the prodrug NPs is the class-based microfluidics chip, which was manually fabricated; therefore, the uniformity of the chip is critical to minimize the batch-to-batch variation. We proved that the opening of the chip’s inner capillary had minor effects on the morphology of the as-prepared NPs [14]. However, we think that the quality of the microfluidics chip can be further improved by industrial manufacturing technologies. Kotz et al. created transparent fused silica glass components using stereolithography three-dimensional (3D) printers at resolutions of a few tens of micrometers [24]; others use 3D-fabricated arbitrarily suspended hollow microstructures in transparent fused silica glass [25]. Inspired by the above studies, we are confident that the microfluids chip can be produced with 3D printing strategy to improve quality control.

The contribution of EPR to the internalization of NPs into solid tumors remains controversial. Although Shrey et al. reported that up to 97% of nanoparticles enter tumors using an active process through endothelial cells [26], others discovered that EPR produces an effect in more than 87% of human renal tumors [27]. Herein, the NPs accumulation in cancer cell was clearly observed, implying that EPR plays a critical role in cancer cell growth inhibition. The efficient accumulation of NPs in the cancer cell line will reduce the side effects of chemotherapy. The addition of reducing agent DTT significantly improved the potency of parent drugs PTX and CPT. A possible explanation is that both amide bond hydrolysis and disulfide bond breakage are involved in this process. In the beginning, DTT does not easily penetrate the prodrug nanoparticles. The CPT is predominantly released by hydrolysis of the ester bond by esterase in the cell in PBS solution. Thereafter, the DTT can easily approach the disulfide bond in the prodrug NPs, due to the resulting swelling. CPT can then be released by breaking the disulfide bond. However, the release profiles of samples with and without DTT differed in the same of toxicity assay, indicating the reduced environment is favorable for liberating the parent drugs.

## 5. Conclusions

To synergistically treat non-small cell lung cancer cell lines, we covalently conjugated CPT and PTX through a disulfide linker to produce CPT-S-S-PTX prodrug. Then, we fabricated the prodrug into NPs using a built-in microfluidics device after the optimization of two parameters: the input concentration of prodrug and I/O flow rate. The as-prepared prodrug NPs had no clear toxicity to healthy cells, but selectively inhibited the cancer. By tracking the prodrug with a special chromophore of CPT, the prodrug showed relatively higher uptake to cancer cells in comparison to healthy cells. As a result, we constructed a prodrug nanoparticle that inhibits cancer cell growth but has limited toxicity to normal cells, while being trackable with a confocal microscope.

## Supplementary Materials

The following are available online at https://www.mdpi.com/1999-4923/12/12/1169/s1, Figure S1: Chromatography spectra of (A) CPT; (B) PTX and (C) DOX-S-S-PTX, Figure S2: Standard curve of (A) CPT; (B) PTX, Figure S3. High resolution mass spectra of (A) PTX-S-S-COOH; (B) CPT-S-S-PTX.
