# Supplementary material for: Self-Assembled Disulfide Bond Bearing Paclitaxel—Camptothecin Prodrug Nanoparticle for Lung Cancer Therapy
Source: Pharmaceutics. 2020 Dec 1;12(12):1169. doi: 10.3390/pharmaceutics12121169 (PMC7760941; doi:10.3390/pharmaceutics12121169)
Supplement: Supplementary file 1 [file pharmaceutics-12-01169-s001.pdf]

pharmaceutics

MDPI

Supplementary Materials: Self-Assembled Disulfide Bond Bearing Paclitaxel– Camptothecin Prodrug Nanoparticle for Lung Cancer Therapy

Jingyan Gao, Xiaodong Ma, Lirong Zhang, Jiaqi Yan, Huaguang Cui, Yuezhou Zhang, Dongqin Wang and Hongbo Zhang

1. High Performance Liquid Chromatography (HPLC) to Identify the Prodrug

Quantification of PTX and DOX was performed by following the method. Gradient analytical HPLC assay was performed on an Agilent 1100 instrument and 20 μL of solution was loaded onto a Waters reverse phase column (250 × 4.6 mm). Acetonitrile (TFA 0.1%): water (TFA 0.1%) (Acetonitrile increase from 5 to 95% with 20 minutes) was eluted at a flow rate of 1 mL/min and all the compounds were detected at 254 nm by UV detector (UV-975, Jasco). A gradient of 5-95% solvent B over 20 min for a 25 min run time was used, to determine that the aforementioned time of starting materials CPT, PTX, and CPT-S-S-PTX was 13.285 min, 17.950 min, and 20.591 min, respectively.

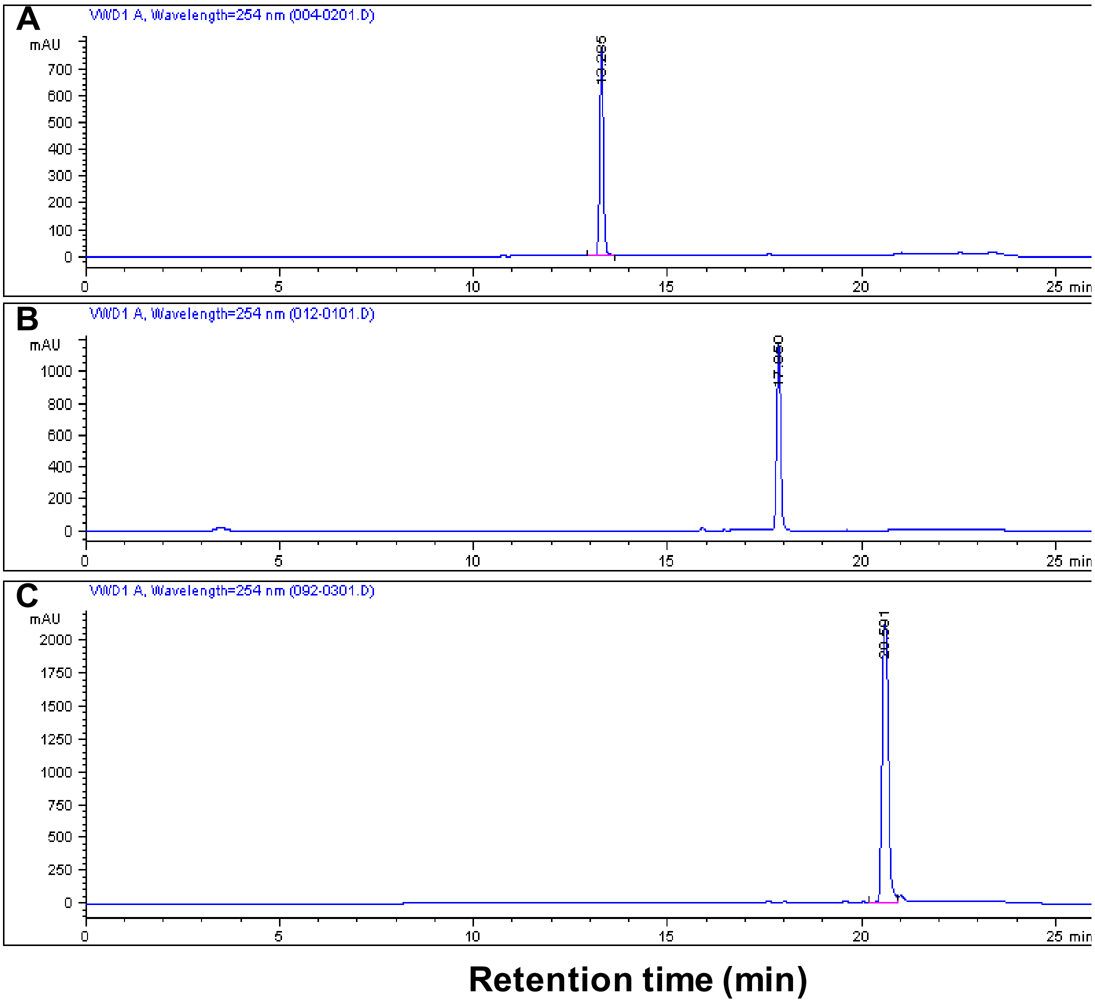

Figure S1. Chromatography spectra of (A) CPT; (B) PTX and (C) DOX-S-S-PTX.

2. Standard Calibration Curves of Parent Drug

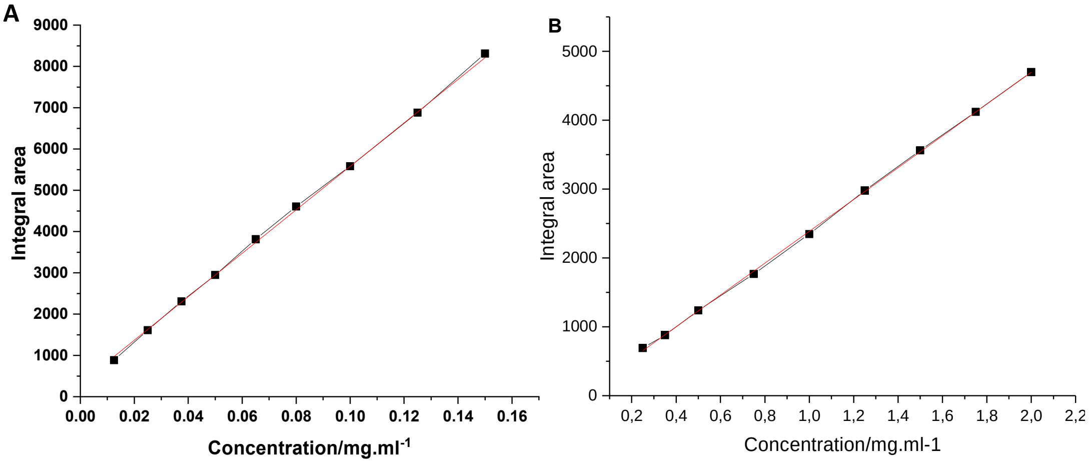

Figure S2. Standard curve of (A) CPT; (B) PTX.

The equation of standard curves for CPT:

$$y = 316.0409 + 52645.47567x \quad R^2 = 0.99874 \tag{1}$$

where y is UV absorption integral of CPT at 254 wavelengths; x is the concentration of DOX.

The equation of standard curves for PTX:

$$y = 75.24836 + 2310.54168x \quad R^2 = 0.99983 \tag{2}$$

where y is UV absorption integral of PTX at 254 wavelengths; x is the concentration of PTX.

The peak areas corresponding to individual compounds were integrated by comparison with external standard calibration curves. Validation of quantitative method was performed with samples for three times. The results of the three injections from the same samples showed similar retention time.

3. Q-TOF(HRMS)

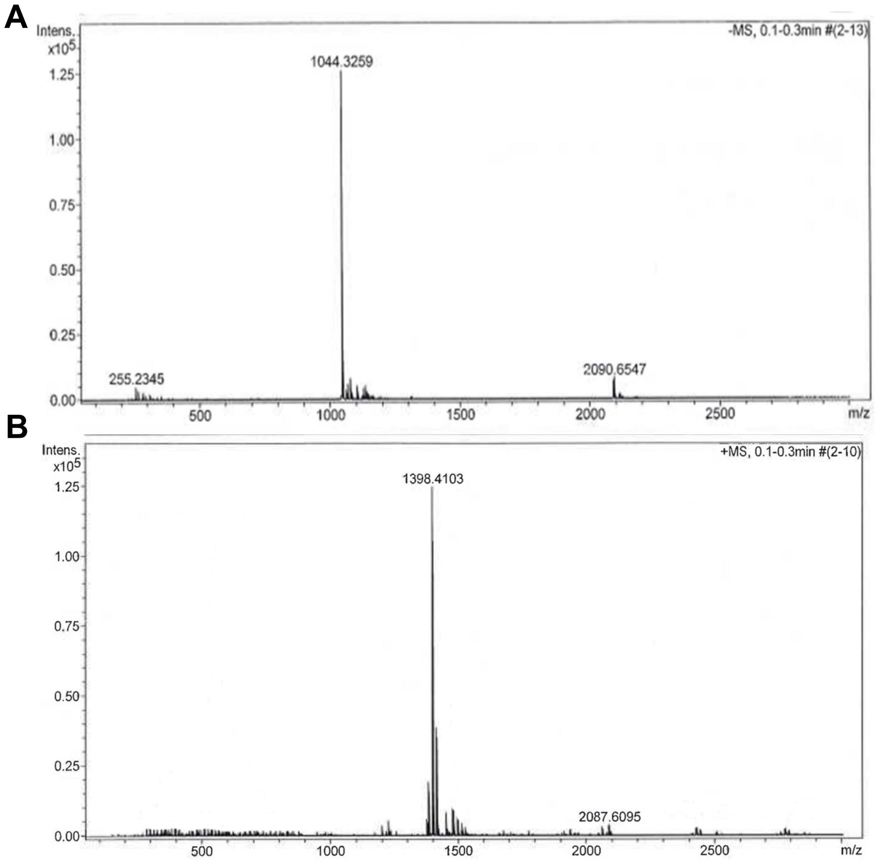

Figure S3. High resolution mass spectra of (A) PTX-S-S-COOH; (B) CPT-S-S-PTX.
